# A curated benchmark dataset for molecular identification based on genome skimming

**DOI:** 10.1038/s41597-025-05230-2

**Published:** 2025-05-29

**Authors:** Renata C. Asprino, Liming Cai, Yujing Yan, Peter J. Flynn, Lucas C. Marinho, Xiaoshan Duan, Christiane Anderson, Goia M. Lyra, Charles C. Davis, Bruno A. S. de Medeiros

**Affiliations:** 1 Programa de Pós-Graduação em Botânica, Universidade Estadual de Feira de Santana, Feira de Santana, Bahia Brazil; 2 Botany, School of Natural Sciences, Trinity College Dublin, Dublin, Ireland; 3 Department of Organismic and Evolutionary Biology, Harvard University Herbaria, Harvard University, Cambridge, Massachusetts 02138 USA; 4 Department of Integrative Biology, The University of Texas at Austin, Austin, Texas 78712 USA; 5 University of Florida, Gainesville, USA; 6 Departamento de Biologia, Universidade Federal do Maranhão, São Luís, Maranhão Brazil; 7 College of Forestry, Northwest Agriculture & Forestry University, Yangling, 712100 Shaanxi China; 8 University of Michigan Herbarium, Ann Arbor, Michigan 48108 USA; 9 Departamento de Biologia Vegetal, Universidade do Estado do Rio de Janeiro, Rio de Janeiro, Brazil; 10 Field Museum of Natural History, Chicago, Illinois 60605 USA; 11 Smithsonian Tropical Research Institute, Panama City, Panama; 12 Museum of Comparative Zoology, Harvard University, Cambridge, Massachusetts 02138 USA

**Keywords:** Taxonomy, Classification and taxonomy

## Abstract

Genome skimming is a promising sequencing strategy for DNA-based taxonomic identification. However, the lack of standardized datasets for benchmarking genome skimming tools presents a challenge in comparing new methods to existing ones. As part of the development of varKoder, a new tool for DNA-based identification, we curated four datasets designed for comparing molecular identification tools using low-coverage genomes. These datasets comprise vast phylogenetic and taxonomic diversity from closely related species to all taxa currently represented on NCBI SRA. One of them consists of novel sequences from taxonomically verified samples in the plant clade Malpighiales, while the other three datasets compile publicly available data. All include raw genome skim sequences to enable comprehensive testing and validation of a variety molecular species identification methods. We also provide the two-dimensional graphical representations of genomic data used in varKoder. These datasets represent a reliable resource for researchers to assess the accuracy, efficiency, and robustness of new tools to varKoder and other methods in a consistent and reproducible manner.

## Background & Summary

Genome skimming has become a versatile tool for biodiversity science, with broad-reaching applications spanning phylogenetics to species identification^1–5^. Low-coverage genomic sequencing facilitates the assembly of both traditional DNA-marker barcodes^6^ as well as barcodes that include entire organellar genomes and many nuclear ribosomal genes^3,7^. These DNA barcodes are important for many uses, such as authenticating plant species of human use^8,9^. One major advantage of genome skimming protocols in relation to PCR-based approaches is that they are robust to DNA quality, being ideal for specimens from Natural History collections, which may present degraded DNA^10^. More recently, genome skimming data are being applied for innovative assembly- and alignment-free species identification^1,11,12^. A large number of methods^1,12–20^ have been developed to apply molecular identification and, typically, their accuracy and efficiency are evaluated with a custom dataset. The customized nature of such datasets is potentially problematic because the success of a given method may be dataset-dependent.

We believe this problem can be solved with a readily accessible and well-annotated benchmark dataset. Specifically, the use of benchmarking datasets plays an essential role in both testing novel methods and guiding the improvement of existing methods by allowing unbiased method comparison and reduced errors due to data variation^21,22^. Benchmarking datasets also help to identify and address potentially confounding variables affecting the performance of different methods. These datasets are of widespread interest to computer scientists across different disciplines, each addressing unique challenges within their respective fields. Fields as diverse as text transcription^23,24^, medical diagnostics^25,26^, and bioinformatics^27,28^ have invested in developing standardized datasets to facilitate the validation and comparison of analytical tools.

A few such datasets also exist in the field of genomics, notably targeted to the tasks of orthology, variant and function prediction. For the former case, OrthoBench^29,30^ has emerged as the standard benchmarking dataset against which orthogroup inference algorithms have been tested for over a decade. The major benchmark dataset for variant prediction is VariBench^21^, which supports the development and evaluation of computational methods for interpreting genetic variants, crucial for improving disease diagnosis and understanding genetic differences across various applications. Finally, there is a newly curated collection of benchmark datasets for genomic functional sequence classification in humans, mice, and roundworms^22^, facilitating the development and evaluation of machine learning models predicting function from DNA sequence data. These models play a crucial role in interpreting vast amounts of genomic data, particularly in human genome investigations, and facilitate discoveries in genetics that have significant implications for medicine and other biological fields.

Another critical challenge in biodiversity and genomic science is the development of DNA-based taxonomic identification methods. In this case, however, we lack a publicly available benchmark dataset similar to those described above. As part of developing **varKoder**, a new method of DNA-based taxonomic identification based on low-coverage genomic reads^1^ (i.e., genome skimming), we have created a number of curated datasets for organisms spanning different taxonomic ranks and phylogenetic depths, from closely related populations, species, to all taxa represented on the NCBI Sequence Read Archive (SRA, https://www.ncbi.nlm.nih.gov/sra/).

To facilitate future comparisons of emerging DNA barcoding methods, here we provide these datasets with metadata and instructions for data access. These datasets are useful for both conventional DNA barcodes^31–35^ and alternative methods that rely on low-coverage genomic sequencing (i.e., DNA signatures^1,36^). They include accession numbers for raw reads that can be applied to any genome skimming method, and the image representations of these genomes that were used in varKoder development, to allow full reproducibility. These data will enable future comparisons to our newly developed approach using the same data that we applied for testing. The datasets made available in this data descriptor include the following: (1) newly sequenced and expert-curated low-coverage whole genome sequencing for species in the flowering plant clade Malpighiales, spanning divergences from closely related species to families, and with samples labeled at species, genus and family levels (2) species-level datasets for plants, animals, fungi and bacteria obtained from the literature, and samples labeled at the species level or below (3) a dataset including all eukaryotic families from the NCBI SRA, labeled at the family level and (4) a dataset with all taxa available from the NCBI SRA, labeled with their complete taxonomic classification. The newly sequenced Malpighiales data was used to extensively compare varKoder^1^ to alternative species identification tools relying on low-coverage genome sequencing, including Skmer^12^, iDeLUCS^37^, and conventional barcodes assembled with PhyloHerb^38^. The other datasets have been used to test varKoder performance in different contexts, some of them outside the domain of existing methods. For example, neither conventional barcodes or Skmer can be applied to all taxa on NCBI SRA. Metrics and comparisons for these methods are detailed in de Medeiros *et al*.^1^.

## Methods

**Fig. 1:**
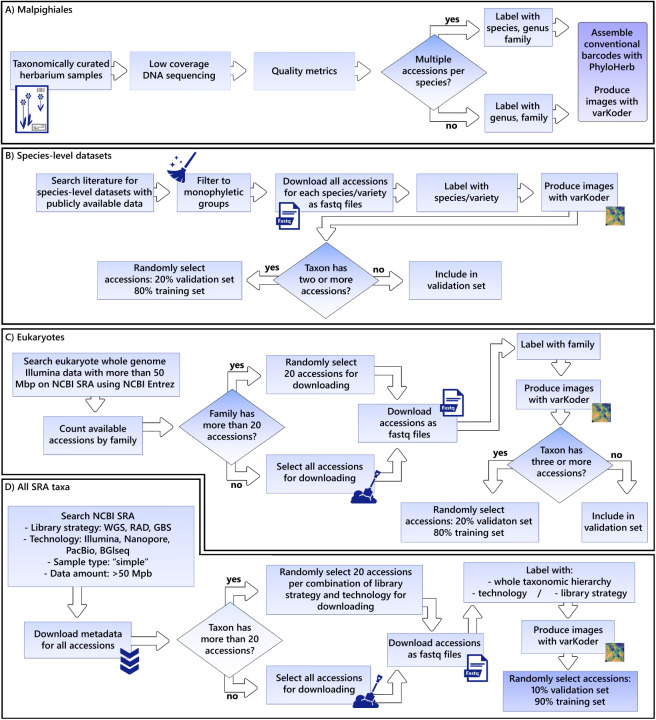
An overview of data collection and the workflow used to create and curate each dataset. The datasets were compiled from newly generated sequences or from publicly available data, following filtering and processing steps shown here.

Each of the four datasets includes sequencing data and image representations derived from them (i.e., varKodes and ranked frequency chaos game representations^1^). Figure 1 provides an overview of the sampling strategy for each dataset and the workflow used to assemble them.

### Taxon sampling with varying phylogenetic depths

#### Malpighiales dataset

This newly generated dataset tests hierarchical classification from species to family level in plants. Plants exhibit notoriously complex genomic architectures^39^ that challenge the performance of conventional DNA barcoding^40^, rendering them a good test case for molecular identification tools. This dataset includes three flowering plant families, all members of the large and morphologically diverse order Malpighiales^41–43^: Malpighiaceae, Elatinaceae, and Chrysobalanaceae. See below for laboratory methods applied for collecting these newly generated sequences.

The Malpighiaceae data are the most taxonomically sampled and include 287 accessions representing 195 species, which were sampled from 277 herbarium specimens and ten silica-dried field collections. Among these data, the genus *Stigmaphyllon* was comprehensively sampled to build, validate, and test identification methods at shallower phylogenetic depths. A total of 100 *Stigmaphyllon* samples were collected, including 10 accessions per species across 10 species. One main advantage of sampling *Stigmaphyllon* is that its taxonomy has been extensively revised, resulting in a diverse and clearly classified set of samples^44,45^. Moreover, the *Stigmaphyllon* clade represents a wide array of divergence times that span distantly- (34.1 Myr) to very closely-related (0.6 Myr) species^1,46^.

The focus for the remainder of the sampling in Malpighiales (Malpighiaceae, Chrysobalanaceae, and Elatinaceae) is to identify a given sample to genus or family. In this case, among the non-*Stigmaphyllon* samples we included 3–9 species per genus representing 30 genera of Malpighiaceae, eight of Chrysobalanaceae, and one of Elatinaceae. Each sample representative was labeled with its corresponding genus and family identification.

#### Species- and subspecies-level datasets

To test shallow-level classification at species or lower taxonomic ranks, we compiled four datasets from publicly available genome skimming data from the NCBI SRA using NCBI Entrez. These datasets include one bacterial species and one genus each from plants, animals, and fungi.

First, we included a dataset from *Mycobacterium tuberculosis*, the species of pathogenic bacteria that causes tuberculosis. The bacterial set consisted of clinical isolates from five distinct, monophyletic lineages of *M. tuberculosis* (1.2.2.1, 2.2.1.1.1, 3.1.2, L4.1.i1.2.1, and L4.3.i2) with seven clinical isolates per lineage, totaling 35 samples. This dataset enables testing identification tools on an extremely recently diverged, clinically relevant bacterial lineage^47^. This dataset of clinical isolates from human-adapted lineages exhibited 99.9% sequence similarity despite key differences in phenotypes, including drug resistance, virulence, and transmissibility^47^. *Mycobacterium tuberculosis* has diversified quite rapidly in humans, with nine monophyletic lineages. Divergence time estimates for the most recent common ancestor of *M. tuberculosis* are <6,000 years ago^48^. The validation set included 3–6 different samples from the five training lineages as well as 1–4 samples from lineages not included in the training set (2.1, 4.10.i1, and 4.6.2.1.1.1.1), totaling 25 validation samples.

For plants, we included a dataset from a well-delineated clade of mycoheterotrophic orchids^49^ (genus *Corallorhiza*), that allows for assessing the infraspecific taxa variation. *Corallorhiza striata* includes several well-known and easily identifiable varieties. For the *Corallorhiza* training set, we included five species (or varieties) with at least five samples per species/variety (for *C. bentleyi*, *C. striata* var. *involuta*, *C. striata*), except for *C. striata* var*. vreelandii* and *C. striata* var*. striata*, for which we included six and seven samples each, respectively, totaling 28 samples. The validation set included 2–11 different samples from three of the five training species/varieties (*C. striata*, *C. striata var. striata*, and *C. striata* var. *vreelandii*) as well as one sample from *C. trifida* which was not included in the training set, totaling 18 validation samples.

For animals, we assembled a *Bembidion* beetle dataset, which includes well-known closely-related cryptic species that were the target of extensive low-coverage whole-genome sequencing^50,51^. The training set included five samples for each of five species including *B. breve*, *B. ampliatum*, *B. lividulum*, *B. saturatum*, and *B. testatum*, totaling 25 samples. The validation set included 1–4 different samples from the five training species as well as from species not included in the training set including *B. aeruginosum*, *B. curtulatum*, *B. geopearlis*, *B. neocoerulescens*, and *B. oromaia*, totaling 18 samples.

For fungi, we used *Xanthoparmelia*, a lichen-forming fungal genus whose species are poorly understood and which often form paraphyletic species groupings^52^. Samples for *Bembidion, Corallorhiza*, and *Mycobacterium tuberculosis* isolates all formed monophyletic groups, whereas *Xanthoparmelia* species did not. Since the *Xanthoparmelia* species were paraphyletic, we subsampled only monophyletic groups for model training. In this case, four species included three samples per species (*X. camtschadalis*, *X. mexicana*, *X. neocumberlandia*, and *X. coloradoensis*) and one species included five samples (*X. chlorochroa*) for the training set, totaling 17 samples. One potential confounding factor is that *Xanthoparmelia* is a lichen-forming fungus and thus genome-skim data represents a chimera of fungal and algal genomes representing both partners in this unique symbiosis. Species of the algal symbiont *Trebouxia* are flexible generalists across fungal *Xanthoparmelia* species. Since these genome skims are a mix of both algal photobiont and fungus, we expect this to be a challenging identification problem because of the more generalist nature of *Trebouxia*^53^. The validation set included 1–3 different samples from the five training species as well as one sample from species not included in the training set including *X. maricopensis*, *X. plittii*, *X. psoromifera*, *X. stenophylla*, *X. sublaevis*, totaling 15 validation samples.

#### Eukaryote family-level dataset

We retrieved DNA sequencing data from the NCBI SRA on March 7, 2023 using NCBI Entrez, filtering for whole genome sequencing data with random library selection from Eukaryotes (taxid:2759), requiring fastq file availability and DNA as biomolecular type. For each record, we collected taxonomic information using NCBI’s Taxonomy database to retrieve family and kingdom classification. Records were filtered to include only those sequenced on the Illumina platform with more than 50 million sequenced bases. To ensure balanced representation across taxa, we randomly selected one sequencing run per taxon, and then randomly selected up to 20 taxa per family. For each sample, we used fastq-dump (https://hpc.nih.gov/apps/sratoolkit.html) to download 500,000 reads, skipping the first 10,000 reads for each accession. The resulting dataset comprises 8,222 accessions, including families of animals (5,642 accessions, 1,426 families), plants (2,705 accessions, 401 families) and fungi (1,572 accessions, 363 families).

#### All-taxa dataset

We retrieved DNA sequencing data from the NCBI SRA using NCBI Entrez on January 9, 2024 and the following criteria: (1) fastq file availability, (2) DNA as biomolecular type, (3) library strategies limited to Genotyping by Sequencing (GBS), Restriction site Associated DNA sequencing (RAD-Seq), or Whole Genome Sequencing (WGS), (4) sample type “simple”, (5) sequencing platform including Illumina, Oxford Nanopore, PacBio SMRT, or BGISEQ, (6) more than 50 million sequenced bases. For each record, we collected taxonomic information of the full taxonomic hierarchy using NCBI’s Taxonomy database. To ensure balanced representation across taxa and methodologies, we randomly selected up to 20 records for each unique combination of taxonomic ID, library strategy, and sequencing platform to avoid overrepresentation of model species such as humans, mice, and *Escherichia coli*. For each sample, we calculated a target number of reads estimated to yield 60 million bases from the SRA record metadata, approximately three times the amount needed for 20 million bases of quality-filtered sequence. We then used fastq-dump to download that number of spots per sample (or at least 10,000 spots, if the estimated number was smaller than that). The resulting dataset includes 253,820 accessions including 28,636 taxonomic labels.

### Laboratory methods for newly generated data

For our newly sequenced Malpighiales data we used total genomic DNA extractions. We isolated total genomic DNA from 0.01–0.02 g of silica-dried leaf material or, more commonly, herbarium collections using the Maxwell 16 DNA Purification Kit (Promega Corporation, Inc., WI, USA) and quantified it using the Qubit 4.0 Fluorometer (Invitrogen, CA, USA), with the Qubit dsDNA HS Assay Kit (Thermo Fisher Scientific, Inc., MA, USA). Our sampling of herbaria followed the guidelines for effective and ethical sampling of these resources outlined by Davis *et al*.^54^. Genomic libraries were prepared using ca. 70 ng of genomic DNA where possible, using 1/8 reactions of the Kapa HyperPlus Library Preparation Kit (Roche, Basel, Switzerland). Libraries were indexed by using the IDT for Illumina TruSeq DNA unique dual 8 bp barcodes (Illumina Inc., San Diego, CA, USA) or the Nextflex-Ht barcodes (Bioo Scientific Corporation, TX, USA) for multiplexing up to 384 samples per sequencing lane. For library preparation, the genomic DNA was sheared by enzymatic fragmentation to 350–400 base pairs (bp). Libraries’ concentrations were verified with the Qubit 4.0 Fluorometer, using the Qubit dsDNA HS Assay Kit (Invitrogen, CA, USA), and average sizes of DNA fragments were verified with the High Sensitivity HSD1000 ScreenTape Assay in the 2200 TapeStation (Agilent Technologies, Waldbronn, Germany). Libraries were diluted into 0.7 nM or 1.0 nM and pooled together. We used Real-Time PCR (BioRad CFX96 Touch, BioRad Laboratories, Hercule, USA) with the NEBNext Library Quant Kit (New England Biolabs, Ipswich, USA) for verifying the final concentration of the libraries’ pools. Sequencing of libraries was conducted using the Illumina Hi-Seq 2500 or the Illumina NovaSeq 6000 (Illumina Inc., San Diego, CA, USA) for 125 bp or 150 bp pair-ended reads, at The Bauer Core Facility at Harvard University, MA, USA.

### Extracting conventional barcodes from genome skimming data

For the Malpighiales dataset, we assembled conventional barcodes. To recover the traditional plant barcodes *rbc*L, *mat*K, *trn*L-F, *ndh*F, and ITS from our Malpighiales genome skim data, we applied GetOrganelle v1.7.7.0^55^ and PhyloHerb v1.1.1^38^ to automatically assemble and extract these DNA markers, respectively. Briefly, the complete or subsampled genome skim data were first assembled into plastid genomes or nuclear ribosomal regions using GetOrganelle^55^ with its default settings. Next, PhyloHerb^38^ was applied to extract the relevant barcode genes using its built-in BLAST database.

### Creation of varKode and CGR images from genome skimming data

**Fig. 2:**
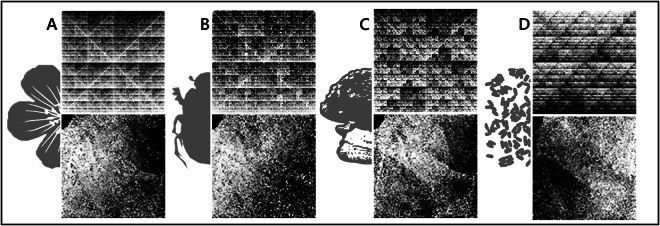
Demonstration of the two types of image representations of the genome signature included in our datasets. Examples of rfCGRs (top) and varKodes (bottom) are shown for four different clades: plants (**A**), animals (**B**), fungi (**C**), and bacteria (**D**). rfCGRs are larger images, and their relative sizes are shown to scale. In each case, both images were produced from the same sequence data. (**A**) Local ID 1089 (plant, *Triaspis hypericoides*) (**B**) SRA Accession SRR15249224 (beetle, *Mesosa* sp.). (**C**) SRA Accession SRR15292413 (fungus, *Amania* sp.). (**D**) SRA Accession SRR2101396 (Bacteria, *Mycobacterium tuberculosis*).

In addition to raw sequence data, we provide image representations of the genome signature (Fig. 2) implied by these data for all samples included here. See our companion paper^1^ for details on how these images are generated. In all cases, pixels in these images represent individual k-mer sequences. Brightness represents the frequency of a k-mer, transformed to ranks and digitized to 8 bits. The two kinds of representation provided differ in how k-mers are mapped to pixels. VarKodes are a compact representation in which k-mer counts and their reverse complements are combined. The mapping of k-mers to pixels in an image attempts to place more similar k-mers closer together in the image space. Ranked frequency chaos game representation (rfCGR) images are similarly produced, but the mapping of k-mers to pixels follows the chaos game representation^56^. rfCGRs present a fractal pattern, while varKodes generally present gradients spanning the whole image. In both cases, we used the “varKoder image” command to generate varKodes, and then used “varKoder convert” to generate rfCGRs from these varKodes. In all cases, we used k-mers of size seven, which were determined to yield optimal balance between classification accuracy and computing effort^1^. These k-mer counts were used to generate images and we normalized counts by ranking and then rescaling and quantizing ranks to integer numbers ranging from 0 to 255, which are the brightness levels supported by a png image. All images are saved in png format, including built-in exif metadata with the labels assigned to each sample. After producing images, we split datasets into training and validation sets. The following specific settings have been used for each dataset described below.

#### Malpighiales

varKodes have been produced from data amounts varying from 500Kbp to 200 Mbp and k-mer size of 7. We applied leave-one-out cross-validation in all tests following de Medeiros *et al*.^1^, so the dataset has not been split into training and validation sets. All accessions have been labelled with their genus and family identification. For species in the genus *Stigmaphyllon*, we additionally labeled accessions with their species identity.

#### Species- and subspecies-level datasets

varKodes have been produced from data amounts varying from 500 Kbp to the maximum amount of data available for each accession and k-mer size of 7. All accessions have received a single label: their species or variety name. For species or varieties represented by at least four accessions, we randomly chose 20% of the accessions for the validation set (with a minimum of 1) and 80% for the training set. For species or varieties with three or less accessions, they were only included in the validation set, to test whether a multi-label model correctly predicted no labels for that accession.

#### NCBI SRA eukaryotes

varKodes have been produced from data amounts varying from 500Kbp to 10Mbp and k-mer size of 7. All accessions have received a single label: their family name. For families represented by at least three accessions, we randomly chose 20% of the accessions for the validation set (with a minimum of 1) and 80% for the training set. Families with less than two accessions were only included in the validation set, to test whether a multi-label model correctly predicted no labels for that accession.

#### NCBI SRA all-taxa

varKodes have been produced from data amounts varying from 500Kbp to 20Mbp and k-mer size of 7. All accessions received multiple labels, including: (1) all NCBI taxonomy IDs related to that accession (i.e., the full taxonomic hierarchy, as separate labels), (2) the library strategy, and (3) the sequencing platform. We randomly selected 10% of the accessions for the validation set, regardless of their labels. Next, we removed from the validation set any labels not present in at least one accession in the training set.

## Data Records

The dataset is available at Harvard Dataverse and the NCBI Sequence Read Archive. The Harvard Dataverse repository^57^ includes metadata tables, processed conventional DNA barcodes, and DNA signature images (varKodes and rfCGRs). New sequences (i.e., Malpighiales) have been uploaded to NCBI SRA under SRP479128^58^. All remaining sequence data were already publicly available on NCBI SRA and can be retrieved from the accession numbers in the metadata tables. The complete dataset comprises four major components, summarized below. See Methods for details on each dataset composition.

To maximize the utility of our datasets for benchmarking molecular identification tools, we provide comprehensive metadata for each sample. The metadata is organized in a consistent format across all datasets to enable easy comparison and reuse in future investigations. Each dataset—Malpighiales, Species and subspecies-level (*Bembidion* beetles, *Corallorhiza* orchids, *Xanthoparmelia* fungi, *Mycobacterium* tuberculosis), Eukaryote families and All SRA taxa—includes a metadata table detailing the raw sequencing data for each sample, with taxonomic-, sequencing-, and sample-related information. All datasets share 17 common metadata fields (Table 1). The Malpighiales dataset, the only one containing new sequence data, includes five additional fields that provide more specific details on voucher information (Table 2). The metadata is provided in the Harvard Dataverse repository^57^.

**Table 1:** Description of common metadata fields for all datasets.

Field	Description
**SRA_Run_ID**	The unique identifier for the run in the NCBI SRA.
**Local_ID**	A unique identifier assigned to each sample as used in de Medeiros *et al*.^1^. This serves as a local reference for linking metadata, sequence data and images.
**Tax_ID**	The taxonomic identifier associated with the organism, as per the NCBI taxonomy.
**Taxon**	The scientific name of the organism from which the sample was derived.
**Taxonomy_Superkingdom**	Taxonomic classification at the Superkingdom level (i.e., Eukaryota, Bacteria, Viruses or Archaea).
**Taxonomy_Kingdom**	Taxonomic classification at the Kingdom level.
**Taxonomy_Family**	Taxonomic classification at the Family level.
**BioSample_ID**	The unique identifier for the sample in NCBI’s BioSample database, linking to additional metadata.
**Download_Path**	URL to reads on the NCBI SRA.
**Library_Strategy**	Sequencing strategy (e.g., WGS, RAD-Seq).
**Library_Source**	DNA source (i.e., genomic DNA or metagenomic).
**Library_Layout**	Configuration of sequencing reads: SINGLE (single-end) or PAIRED (paired-end).
**Seq_Platform**	Sequencing Platform, such as Illumina, PacBio, Oxford Nanopore, etc.
**Seq_Model**	Sequencing Instrument (e.g., Illumina NovaSeq 6000).
**Size_MB**	Amount of SRA sequencing data in millions of base pairs (MB).
**Labels**	All the labels assigned to a given accession, combined as a string separated by semicolon.
**Set**	Set in de Medeiros *et al*.^1^. For the Malpighiales dataset, this column has empty values since samples were evaluated with cross-validation. For other datasets: “train” for training set, “valid” for validation set and “valid_notrain” for accessions used in validation but with taxonomic labels not included in the training set, to test for false positives.

**Table 2:** Description of additional metadata fields exclusive in the Malpighiales dataset.

Field	Description
**Taxonomy_Genus**	Genus to which the sample belongs, to support identification to genus level.
**Voucher**	Information on the collector and the collection number, which links the sample to its voucher specimen.
**Collector**	The name of the individual(s) responsible for collecting the specimen.
**CollectorID**	The specific number associated with the collector’s collection for this sample.
**Collection**	The acronym of the collection where the herbarium voucher of the sample is deposited.

### Malpighiales

This dataset contains 287 newly sequenced accessions from three families in the order Malpighiales. This includes families Malpighiaceae (251 accessions representing 31 genera), Elatinaceae (6 accessions for 1 genus), and Chrysobalanaceae (30 accessions for 8 genera). Malpighiaceae includes *Stigmaphyllon* with the most comprehensive species sampling: 10 species and 10 accessions sampled per species. *Stigmaphyllon* accessions are labeled with species, genus and family. All other accessions are labeled with genus and family. This dataset is used for benchmarking molecular identification tools from species to family levels under a realistic scenario of uneven diversity and sequencing effort. The data provided includes raw sequencing data, processed conventional barcodes (*rbc*L, *mat*K, *trn*L-F, *ndh*F, and ITS), and image representations (varKodes and rfCGRs).

### Species- and subspecies-level datasets

This is composed of four datasets from published data of four clades – *Bembidion* beetles (43 accessions from 10 species), *Corallorhiza* orchids (46 accessions from 6 species/varieties), *Xanthoparmelia* fungi (32 accessions from 10 species), and *Mycobacterium* bacteria (60 accessions from 8 lineages). In each case, we include raw sequencing data and image representations. These datasets are suitable for benchmarking species-level identification, as well as variety, strain, or subspecies.

### Eukaryote families

We compiled a dataset for identifying eukaryote families from the NCBI Sequence Read Archive. This includes 9,910 accessions from 2,182 families of animals, plants and fungi. Of these, 861 families (517 Metazoa, 197 plants, 147 fungi), represented by 8,222 accessions, had at least three accessions available and were included in the training set. We include sequence data and image representations. This dataset serves to benchmark family-level identification tools at a large scale.

### All SRA taxa

This is the largest dataset compiled from the NCBI Sequence Read Archive, containing data including all the taxonomic hierarchy and multiple sequencing methods (253,820 accessions including 28,636 taxonomic labels, three labels for library strategy, and four labels for sequencing platform). We include sequence data and image representations. This is the largest and most heterogeneous dataset provided here, benchmarking identification at all taxonomic levels across different sequencing methodologies.

For raw sequence data, we provide accession numbers to NCBI SRA runs. These can be downloaded in conventional formats (such as fastq) using the SRA toolkit (https://github.com/ncbi/sra-tools).

Processed conventional barcodes are provided as fasta files. Each fasta file is named after the gene region represented and includes individual sequences named after the SRA accession number.

Image representations are provided as png images. These images follow a file name convention that is interpreted by **varKoder** and include information about accession number, k-mer size, type of representation and amount of DNA sequence data used to produce the image: “[local_ID]@[sequence base pairs] + [representation] + k[k-mer size].png”. For example, the file “SRR9036258@00010000 K + varKode + k7.png” represents accession with local ID SRR9036258, 10 Mbp (i.e., 10,000 Kbp) of sequence data, varKode representation and k-mer size of 7. Labels associated with accession can be found in the metadata tables and also as image metadata contained in the png file. **varKoder** is able to read this image metadata, and it is also visible through general purpose programs that handle image metadata, such as exiftool (https://exiftool.org).

## Technical Validation

We measured sequencing success using various quality metrics for raw reads and the plastid assemblies produced from them. These include the sequencing yield, percentage of bases with a quality score above 30, average GC content of the raw sequencing output, whether plastid assemblies were complete and the assembly size. Raw read metrics were estimated with fastp v. 0.23.2^59^ and assembly metrics with GetOrganelle. These metrics were calculated for the newly sequenced data of Malpighiales’ representatives to ensure robustness and reliability of the sequencing results. A summary of these metrics are provided in Table S1.

We have not further validated sequences that were already publicly available. In that case, we used data as downloaded from NCBI following the filters specified in Methods.

## Usage Notes

See de Medeiros *et al*.^1^ for a complete account of how these datasets have been used to develop and test varKoder. NCBI accession numbers can be used to download associated sequence data with the SRA toolkit (https://github.com/ncbi/sra-tools). Conventional barcode sequences in the fasta format can be used for sequence alignment and search. varKode and rfCGR images can be used as input to varKoder or other programs processing images in the PNG format. Conventional barcode sequences and PNG images can be found in the Harvard Dataverse repository^57^ accompanying this article.

## Supplementary information

Supplementary Information

## Data Availability

The code used to retrieve and process sequence data used here is available in a github repository (https://github.com/brunoasm/varKoder_development), archived in FigShare (10.6084/m9.figshare.8304017)^60^. The source code for varKoder, which can process sequence data into varKodes and rfGRS, as well as train and use neural networks, is available at https://github.com/brunoasm/varKoder.
