# Supplementary material for: A curated benchmark dataset for molecular identification based on genome skimming
Source: Sci Data. 2025 May 29;12:906. doi: 10.1038/s41597-025-05230-2 (PMC12122930; doi:10.1038/s41597-025-05230-2)
Supplement: Supplementary file 1 — Supplementary Information [file 41597_2025_5230_MOESM1_ESM.docx]

**A curated benchmark dataset for molecular identification based on genome skimming**

Renata C. Asprino, Liming Cai, Yujing Yan, Peter J. Flynn, Lucas C. Marinho, Xiaoshan Duan, Christiane Anderson, Charles C. Davis, and Bruno A. S. de Medeiros

### Supplementary Information

**Table S1**. Quality metrics for newly sequenced data, ordered by assembly size. *SRA run ID:* accession number of NCBI SRA run. *Taxon:* Malpighiales species. *Yield (Mb):* sequencing yield of library. *>= Q30 bases (%):* Percentage of bases with phred quality score above 30. *GC content (%):* average GC content across all reads. *Assembly complete:* whether plastid assembly is complete (X) or fragmented (empty). *Assembly size (Kbp):* Total assembly size. The size of complete plastid genome assemblies from GetOrganelle typically ranges from 150 to 165 kb. In fragmented assemblies, the two 20-kb inverted repeat regions collapse into a single contig, resulting in a significantly reduced assembly size of 120 to 130 kb, but should be considered nearly complete.

| SRA run ID | Taxon | Yield (Mb) | >= Q30 bases (%) | GC Content (%) | Assembly complete | Assembly size (Kbp) |
| --- | --- | --- | --- | --- | --- | --- |
| SRR27295657 | *Dicella aciculifera* | 463 | 95.2 | 35.2 |  | 166.2 |
| SRR27295694 | *Hirtella gracilipes* | 576 | 92.5 | 41.8 | X | 163.0 |
| SRR27295706 | *Hirtella rugosa* | 854 | 93.3 | 50.2 | X | 162.9 |
| SRR27295688 | *Hirtella scabra* | 666 | 90.8 | 42.9 | X | 162.9 |
| SRR27295704 | *Hirtella guatemalensis* | 858 | 93.3 | 49.1 | X | 162.9 |
| SRR27295701 | *Acioa edulis* | 1,464 | 92.6 | 45.2 | X | 162.8 |
| SRR27295703 | *Hirtella americana* | 820 | 92.4 | 42.6 | X | 162.8 |
| SRR27295699 | *Gaulettia parillo* | 702 | 93.4 | 45.4 | X | 162.7 |
| SRR27295684 | *Acioa longipendula* | 796 | 91.9 | 44.2 | X | 162.6 |
| SRR27295745 | *Parinari alvimii* | 745 | 91.0 | 41.7 | X | 162.6 |
| SRR27295696 | *Licania laxiflora* | 709 | 92.6 | 43.6 | X | 162.6 |
| SRR27295687 | *Licania bracteata* | 425 | 92.3 | 46.8 | X | 162.5 |
| SRR27295702 | *Acioa somnolens* | 643 | 92.2 | 41.8 | X | 162.5 |
| SRR27295746 | *Parinari obtusifolia* | 725 | 92.3 | 40.1 | X | 162.5 |
| SRR27295686 | *Licania gracilipes* | 804 | 92.4 | 40.0 | X | 162.4 |
| SRR27295697 | *Parinari nonda* | 609 | 92.8 | 41.2 | X | 162.4 |
| SRR27295685 | *Dactyladenia ndjoleensis* | 363 | 93.1 | 42.7 | X | 162.4 |
| SRR27295744 | *Licania cymosa* | 581 | 90.8 | 40.6 | X | 162.4 |
| SRR27295708 | *Licania cordata* | 637 | 92.6 | 47.5 | X | 162.3 |
| SRR27295693 | *Exellodendron barbatum* | 893 | 92.8 | 42.4 | X | 162.3 |
| SRR27295590 | *Byrsonima dealbata* | 518 | 95.0 | 36.4 |  | 162.3 |
| SRR27295705 | *Dactyladenia scabrifolia* | 1,104 | 91.3 | 46.0 | X | 162.3 |
| SRR27295691 | *Dactyladenia incondere* | 431 | 92.8 | 54.0 | X | 162.3 |
| SRR27295692 | *Gaulettia canomensis* | 893 | 92.2 | 42.0 | X | 162.3 |
| SRR27295698 | *Couepia maguirei* | 1,323 | 91.8 | 44.4 | X | 162.0 |
| SRR27295683 | *Couepia bondarii* | 611 | 93.0 | 44.6 | X | 162.0 |
| SRR27295695 | *Couepia habrantha* | 1,006 | 93.2 | 53.6 | X | 161.9 |
| SRR27295741 | *Couepia oxossii* | 588 | 91.7 | 42.6 | X | 161.9 |
| SRR27295682 | *Couepia uiti* | 1,120 | 93.2 | 40.7 | X | 161.8 |
| SRR27295742 | *Exellodendron gracile* | 688 | 92.8 | 43.0 | X | 161.8 |
| SRR27295531 | *Malpighia ovata* | 446 | 95.3 | 38.0 | X | 161.5 |
| SRR27295533 | *Malpighia diversifolia* | 418 | 94.5 | 37.3 | X | 161.4 |
| SRR27295707 | *Exellodendron gardneri* | 471 | 91.8 | 41.7 | X | 161.1 |
| SRR27295528 | *Heteropterys gentlei* | 422 | 92.5 | 40.5 |  | 160.6 |
| SRR27295661 | *Callaeum coactum* | 114 | 94.9 | 35.1 | X | 160.6 |
| SRR27295530 | *Bunchosia linearifolia* | 131 | 93.9 | 31.8 | X | 160.5 |
| SRR27295743 | *Amorimia septentrionalis* | 352 | 96.8 | 37.0 | X | 160.4 |
| SRR27295631 | *Carolus sinemariensis* | 83 | 94.1 | 37.8 |  | 160.3 |
| SRR27295584 | *Byrsonima morii* | 5711 | 96.4 | 34.6 |  | 160.2 |
| SRR27295690 | *Gaulettia cognata* | 374 | 93.4 | 51.6 |  | 160.1 |
| SRR27295798 | *Microsteira curtisii* | 183 | 95.7 | 34.3 | X | 160.0 |
| SRR27295720 | *Microsteira diotostigma* | 239 | 96.6 | 34.5 | X | 160.0 |
| SRR27295612 | *Stigmaphyllon bonariense* | 1,038 | 91.5 | 42.5 |  | 159.9 |
| SRR27295787 | *Microsteira pluriseta* | 222 | 95.6 | 34.6 | X | 159.9 |
| SRR27295569 | *Stigmaphyllon ellipticum* | 824 | 91.2 | 40.5 |  | 159.9 |
| SRR27295566 | *Stigmaphyllon ellipticum* | 605 | 91.5 | 37.5 | X | 159.8 |
| SRR27295560 | *Stigmaphyllon ellipticum* | 720 | 92.0 | 39.0 | X | 159.8 |
| SRR27295557 | *Stigmaphyllon emarginatum* | 873 | 91.2 | 41.7 | X | 159.8 |
| SRR27295614 | *Stigmaphyllon bonariense* | 368 | 90.6 | 39.4 | X | 159.8 |
| SRR27295564 | *Stigmaphyllon ellipticum* | 795 | 91.6 | 38.7 |  | 159.8 |
| SRR27295734 | *Stigmaphyllon lindenianum* | 785 | 90.8 | 57.3 | X | 159.8 |
| SRR27295628 | *Stigmaphyllon jatrophifolium* | 385 | 91.2 | 41.4 |  | 159.7 |
| SRR27295568 | *Stigmaphyllon ellipticum* | 625 | 90.5 | 42.0 | X | 159.7 |
| SRR27295565 | *Stigmaphyllon ellipticum* | 1,057 | 90.3 | 44.8 | X | 159.7 |
| SRR27295561 | *Stigmaphyllon ellipticum* | 754 | 90.9 | 39.0 | X | 159.7 |
| SRR27295558 | *Stigmaphyllon ellipticum* | 955 | 91.3 | 38.6 | X | 159.7 |
| SRR27295623 | *Stigmaphyllon jatrophifolium* | 626 | 91.5 | 39.9 | X | 159.7 |
| SRR27295563 | *Stigmaphyllon ellipticum* | 906 | 91.2 | 41.1 | X | 159.7 |
| SRR27295629 | *Stigmaphyllon jatrophifolium* | 595 | 91.5 | 41.6 | X | 159.7 |
| SRR27295700 | *Amorimia exotropica* | 164 | 95.7 | 38.2 | X | 159.7 |
| SRR27295567 | *Stigmaphyllon ellipticum* | 801 | 91.9 | 39.6 | X | 159.7 |
| SRR27295622 | *Stigmaphyllon jatrophifolium* | 533 | 91.5 | 39.6 | X | 159.7 |
| SRR27295621 | *Stigmaphyllon jatrophifolium* | 899 | 89.6 | 45.1 | X | 159.7 |
| SRR27295607 | *Stigmaphyllon paralias* | 680 | 91.5 | 39.9 |  | 159.7 |
| SRR27295626 | *Stigmaphyllon jatrophifolium* | 773 | 91.0 | 40.9 | X | 159.7 |
| SRR27295624 | *Stigmaphyllon jatrophifolium* | 754 | 91.7 | 41.3 | X | 159.7 |
| SRR27295675 | *Stigmaphyllon ciliatum* | 547 | 91.7 | 43.3 |  | 159.7 |
| SRR27295549 | *Stigmaphyllon emarginatum* | 909 | 92.5 | 41.9 |  | 159.6 |
| SRR27295548 | *Stigmaphyllon emarginatum* | 885 | 90.9 | 43.9 |  | 159.6 |
| SRR27295634 | *Stigmaphyllon ciliatum* | 609 | 92.3 | 39.9 | X | 159.6 |
| SRR27295625 | *Stigmaphyllon jatrophifolium* | 880 | 91.7 | 40.8 | X | 159.6 |
| SRR27295588 | *Microsteira argyrophylla* | 212 | 95.9 | 32.6 | X | 159.6 |
| SRR27295542 | *Stigmaphyllon puberum* | 699 | 91.9 | 48.5 | X | 159.6 |
| SRR27295545 | *Stigmaphyllon puberum* | 588 | 91.7 | 38.9 | X | 159.6 |
| SRR27295638 | *Stigmaphyllon ciliatum* | 839 | 92.2 | 40.4 | X | 159.6 |
| SRR27295749 | *Mascagnia violacea* | 101 | 90.6 | 38.0 | X | 159.6 |
| SRR27295630 | *Stigmaphyllon ciliatum* | 496 | 90.7 | 40.5 | X | 159.6 |
| SRR27295670 | *Stigmaphyllon bannisterioides* | 540 | 91.2 | 39.5 | X | 159.5 |
| SRR27295679 | *Stigmaphyllon bannisterioides* | 393 | 90.9 | 35.2 | X | 159.5 |
| SRR27295553 | *Stigmaphyllon emarginatum* | 553 | 92.5 | 40.3 |  | 159.5 |
| SRR27295636 | *Stigmaphyllon ciliatum* | 579 | 89.8 | 43.6 |  | 159.5 |
| SRR27295674 | *Stigmaphyllon ciliatum* | 1,192 | 91.0 | 43.8 |  | 159.5 |
| SRR27295672 | *Stigmaphyllon bannisterioides* | 780 | 91.5 | 35.1 | X | 159.5 |
| SRR27295669 | *Stigmaphyllon bannisterioides* | 467 | 89.6 | 38.4 | X | 159.5 |
| SRR27295547 | *Stigmaphyllon paralias* | 713 | 91.8 | 40.8 | X | 159.5 |
| SRR27295587 | *Byrsonima intermedia* | 510 | 96.4 | 36.5 |  | 159.5 |
| SRR27295711 | *Stigmaphyllon bannisterioides* | 772 | 88.5 | 43.2 | X | 159.5 |
| SRR27295738 | *Stigmaphyllon puberum* | 905 | 91.9 | 39.3 | X | 159.4 |
| SRR27295668 | *Stigmaphyllon bannisterioides* | 838 | 91.0 | 36.6 | X | 159.4 |
| SRR27295541 | *Stigmaphyllon puberum* | 662 | 90.5 | 41.5 | X | 159.4 |
| SRR27295667 | *Stigmaphyllon bannisterioides* | 731 | 90.9 | 34.8 | X | 159.4 |
| SRR27295740 | *Stigmaphyllon puberum* | 747 | 91.3 | 51.3 | X | 159.4 |
| SRR27295718 | *Stigmaphyllon bogotense* | 718 | 92.0 | 39.7 |  | 159.4 |
| SRR27295671 | *Stigmaphyllon bannisterioides* | 900 | 91.3 | 38.1 | X | 159.4 |
| SRR27295532 | *Malpighia harrisii* | 295 | 95.9 | 38.3 | X | 159.4 |
| SRR27295716 | *Stigmaphyllon bogotense* | 476 | 91.7 | 41.4 | X | 159.4 |
| SRR27295714 | *Stigmaphyllon bogotense* | 602 | 89.6 | 47.0 | X | 159.3 |
| SRR27295737 | *Stigmaphyllon paralias* | 634 | 91.8 | 39.7 | X | 159.2 |
| SRR27295681 | *Stigmaphyllon paralias* | 748 | 91.6 | 39.8 |  | 159.2 |
| SRR27295722 | *Stigmaphyllon bogotense* | 549 | 91.6 | 40.6 | X | 159.2 |
| SRR27295555 | *Stigmaphyllon emarginatum* | 575 | 91.3 | 41.6 |  | 159.1 |
| SRR27295571 | *Tristellateia greveana* | 167 | 96.3 | 35.9 |  | 159.0 |
| SRR27295633 | *Stigmaphyllon paralias* | 687 | 91.6 | 45.2 |  | 159.0 |
| SRR27295539 | *Stigmaphyllon puberum* | 1,000 | 92.0 | 40.1 | X | 159.0 |
| SRR27295598 | *Bunchosia swartziana* | 409 | 96.9 | 35.3 | X | 158.7 |
| SRR27295758 | *Acridocarpus orientalis* | 140 | 94.5 | 37.2 | X | 158.7 |
| SRR27295788 | *Banisteriopsis irwinii* | 514 | 95.4 | 48.5 | X | 158.5 |
| SRR27295797 | *Tetrapterys heterophylla* | 1119 | 94.4 | 35.4 |  | 158.2 |
| SRR27295536 | *Acridocarpus perrieri* | 307 | 95.0 | 38.9 | X | 158.0 |
| SRR27295660 | *Acridocarpus chevalieri* | 520 | 95.0 | 40.9 | X | 158.0 |
| SRR27295589 | *Diplopterys valvata* | 150 | 81.2 | 36.1 |  | 157.8 |
| SRR27295649 | *Bunchosia decussiflora* | 492 | 95.7 | 34.9 |  | 157.7 |
| SRR27295650 | *Acridocarpus macrocalyx* | 174 | 94.6 | 37.9 | X | 157.7 |
| SRR27295807 | *Aspidopterys wallichii* | 243 | 93.0 | 37.5 | X | 157.6 |
| SRR27295597 | *Galphimia tuberculata* | 91 | 96.4 | 34.7 |  | 156.9 |
| SRR27295662 | *Acridocarpus smeathmannii* | 107 | 94.1 | 37.9 | X | 156.3 |
| SRR27295596 | *Elatine gracilis* | 219 | 92.1 | 43.3 | X | 154.8 |
| SRR27295529 | *Heteropterys quetepensis* | 743 | 95.7 | 38.5 | X | 153.9 |
| SRR27295652 | *Triaspis odorata* | 155 | 85.3 | 39.2 |  | 153.5 |
| SRR27295765 | *Hiptage bullata* | 1071 | 95.3 | 37.4 |  | 152.5 |
| SRR27295790 | *Amorimia camporum* | 232 | 94.0 | 45.8 |  | 149.8 |
| SRR27295526 | *Amorimia concinna* | 586 | 95.9 | 38.1 |  | 143.2 |
| SRR27295808 | *Aspidopterys indica* | 472 | 93.0 | 62.2 |  | 141.2 |
| SRR27295755 | *Mascagnia divaricata* | 138 | 94.3 | 35.2 |  | 136.8 |
| SRR27295592 | *Acridocarpus zanzibaricus* | 123 | 94.7 | 35.9 |  | 136.5 |
| SRR27295656 | *Dicella bracteosa* | 472 | 95.6 | 36.0 |  | 136.4 |
| SRR27295527 | *Acmanthera duckei* | 283 | 95.7 | 39.4 |  | 134.8 |
| SRR27295540 | *Malpighiodes liesneri* | 732 | 94.1 | 33.7 |  | 134.7 |
| SRR27295776 | *Tristellateia ambongensis* | 353 | 94.9 | 38.7 |  | 134.7 |
| SRR27295654 | *Triaspis macropteron* | 92 | 92.7 | 36.7 |  | 134.4 |
| SRR27295750 | *Christianella surinamensis* | 350 | 89.0 | 35.6 |  | 134.1 |
| SRR27295715 | *Stigmaphyllon bogotense* | 488 | 89.8 | 40.9 |  | 134.1 |
| SRR27295620 | *Malpighia lundellii* | 182 | 92.9 | 38.8 |  | 134.0 |
| SRR27295786 | *Banisteriopsis irwinii* | 1955 | 94.4 | 41.6 |  | 134.0 |
| SRR27295595 | *Elatine triandra* | 61 | 92.9 | 45.7 |  | 133.8 |
| SRR27295759 | *Diacidia ferruginea* | 153 | 92.5 | 37.0 |  | 133.5 |
| SRR27295723 | *Stigmaphyllon bogotense* | 893 | 91.6 | 42.4 |  | 133.5 |
| SRR27295809 | *Microsteira ambongensis* | 178 | 95.9 | 33.0 |  | 133.5 |
| SRR27295719 | *Stigmaphyllon bogotense* | 673 | 91.6 | 42.0 |  | 133.5 |
| SRR27295611 | *Stigmaphyllon bonariense* | 680 | 91.8 | 40.2 |  | 133.4 |
| SRR27295585 | *Byrsonima microphylla* | 301 | 92.9 | 39.7 |  | 133.1 |
| SRR27295643 | *Triaspis niedenzuiana* | 90 | 92.6 | 35.7 |  | 133.1 |
| SRR27295677 | *Stigmaphyllon ciliatum* | 721 | 91.7 | 40.9 |  | 133.1 |
| SRR27295721 | *Stigmaphyllon bogotense* | 706 | 91.7 | 41.2 |  | 133.0 |
| SRR27295725 | *Stigmaphyllon paralias* | 476 | 90.1 | 42.4 |  | 133.0 |
| SRR27295663 | *Callaeum nicaraguense* | 58 | 87.6 | 38.6 |  | 132.9 |
| SRR27295710 | *Stigmaphyllon bannisterioides* | 829 | 90.9 | 38.8 |  | 132.9 |
| SRR27295577 | *Elatine alsinastrum* | 152 | 94.4 | 42.5 |  | 132.9 |
| SRR27295712 | *Stigmaphyllon bogotense* | 802 | 91.6 | 41.9 |  | 132.7 |
| SRR27295773 | *Bunchosia cruciana* | 425 | 96.6 | 34.9 |  | 132.6 |
| SRR27295680 | *Stigmaphyllon paralias* | 518 | 91.8 | 40.9 |  | 132.6 |
| SRR27295570 | *Stigmaphyllon bonariense* | 568 | 92.0 | 40.1 |  | 132.4 |
| SRR27295796 | *Tetrapterys jamesonii* | 280 | 95.3 | 42.7 |  | 132.1 |
| SRR27295733 | *Stigmaphyllon lindenianum* | 467 | 91.4 | 42.5 |  | 132.0 |
| SRR27295771 | *Diacidia vestita* | 681 | 95.3 | 43.2 |  | 132.0 |
| SRR27295775 | *Tristellateia cocculifolia* | 187 | 96.1 | 37.8 |  | 132.0 |
| SRR27295546 | *Stigmaphyllon emarginatum* | 599 | 92.1 | 39.7 |  | 132.0 |
| SRR27295732 | *Stigmaphyllon lindenianum* | 657 | 91.4 | 43.1 |  | 131.9 |
| SRR27295644 | *Bronwenia megaptera* | 820 | 94.2 | 37.3 |  | 131.7 |
| SRR27295805 | *Burdachia duckei* | 272 | 93.7 | 39.8 |  | 131.6 |
| SRR27295760 | *Bunchosia veluticarpa* | 309 | 95.6 | 34.9 |  | 131.5 |
| SRR27295717 | *Stigmaphyllon bogotense* | 795 | 91.4 | 43.5 |  | 131.5 |
| SRR27295810 | *Acridocarpus socotranus* | 898 | 90.2 | 37.0 |  | 131.5 |
| SRR27295538 | *Triaspis sapinii* | 162 | 95.5 | 37.5 |  | 131.4 |
| SRR27295709 | *Aspicarpa salicifolia* | 107 | 95.1 | 38.7 |  | 131.2 |
| SRR27295792 | *Acmanthera fernandesii* | 309 | 95.6 | 40.4 |  | 131.2 |
| SRR27295768 | *Triaspis erlangeri* | 510 | 95.0 | 39.9 |  | 130.8 |
| SRR27295726 | *Stigmaphyllon lindenianum* | 744 | 93.0 | 43.3 |  | 130.7 |
| SRR27295576 | *Elatine rubella* | 154 | 94.9 | 39.5 |  | 130.6 |
| SRR27295799 | *Malpighia megacantha* | 257 | 96.0 | 39.0 |  | 130.6 |
| SRR27295666 | *Aspicarpa schininii* | 87 | 94.0 | 35.0 |  | 130.6 |
| SRR27295608 | *Stigmaphyllon bonariense* | 704 | 91.6 | 42.6 |  | 130.5 |
| SRR27295766 | *Cottsia linearis* | 88 | 90.6 | 36.8 |  | 130.4 |
| SRR27295659 | *Callaeum malpighioides* | 122 | 94.8 | 37.1 |  | 130.3 |
| SRR27295800 | *Malpighia emarginata* | 256 | 96.6 | 36.9 |  | 130.2 |
| SRR27295525 | *Aspidopterys glabriuscula* | 144 | 94.1 | 38.8 |  | 129.7 |
| SRR27295777 | *Tristellateia bojerana* | 243 | 96.8 | 38.8 |  | 129.6 |
| SRR27295754 | *Christianella paludicola* | 1533 | 94.5 | 34.0 |  | 129.6 |
| SRR27295616 | *Stigmaphyllon bonariense* | 770 | 91.8 | 39.8 |  | 129.6 |
| SRR27295613 | *Stigmaphyllon bonariense* | 909 | 90.4 | 43.0 |  | 129.6 |
| SRR27295594 | *Elatine hungaria* | 516 | 90.9 | 52.1 |  | 129.6 |
| SRR27295784 | *Camarea hirsuta* | 220 | 92.7 | 39.5 |  | 129.5 |
| SRR27295806 | *Blepharandra cachimbensis* | 245 | 90.3 | 36.8 |  | 129.2 |
| SRR27295651 | *Thryallis parviflora* | 184 | 95.9 | 36.3 |  | 129.2 |
| SRR27295729 | *Stigmaphyllon lindenianum* | 434 | 91.6 | 41.6 |  | 129.1 |
| SRR27295795 | *Christianella multiglandulosa* | 171 | 91.8 | 34.3 |  | 129.1 |
| SRR27295619 | *Stigmaphyllon paralias* | 529 | 91.0 | 41.1 |  | 128.9 |
| SRR27295781 | *Diplopterys lucida* | 677 | 93.5 | 40.6 |  | 128.8 |
| SRR27295804 | *Camarea ericoides* | 215 | 94.1 | 37.8 |  | 128.6 |
| SRR27295544 | *Stigmaphyllon puberum* | 499 | 91.5 | 39.2 |  | 128.6 |
| SRR27295778 | *Tetrapterys skutchii* | 514 | 96.0 | 36.6 |  | 128.6 |
| SRR27295782 | *Dicella julianii* | 188 | 94.8 | 38.6 |  | 128.5 |
| SRR27295579 | *Elatine americana* | 119 | 93.3 | 44.8 |  | 128.4 |
| SRR27295724 | *Stigmaphyllon lindenianum* | 338 | 88.6 | 45.8 |  | 128.2 |
| SRR27295803 | *Cottsia gracilis* | 142 | 95.0 | 38.4 |  | 126.9 |
| SRR27295653 | *Thryallis latifolia* | 389 | 94.5 | 35.3 |  | 126.9 |
| SRR27295646 | *Banisteriopsis calcicola* | 78 | 93.6 | 37.1 |  | 126.7 |
| SRR27295586 | *Byrsonima psilandra* | 762 | 96.0 | 37.1 |  | 125.4 |
| SRR27295791 | *Diacidia kunhardtii* | 153 | 93.0 | 40.2 |  | 125.1 |
| SRR27295575 | *Tetrapterys calophylla* | 155 | 96.1 | 37.6 |  | 124.9 |
| SRR27295580 | *Bunchosia articulata* | 391 | 97.1 | 32.1 |  | 124.9 |
| SRR27295606 | *Diacidia galphimioides* | 104 | 93.0 | 34.2 |  | 124.9 |
| SRR27295658 | *Callaeum psilophyllum* | 105 | 92.8 | 37.2 |  | 124.3 |
| SRR27295600 | *Tristellateia grandiflora* | 379 | 94.3 | 37.1 |  | 124.0 |
| SRR27295599 | *Callaeum johnsonii* | 131 | 93.3 | 35.7 |  | 123.9 |
| SRR27295774 | *Thryallis brachystachys* | 400 | 96.3 | 35.7 |  | 123.1 |
| SRR27295639 | *Mascagnia lugoi* | 436 | 94.1 | 36.6 |  | 123.0 |
| SRR27295664 | *Aspicarpa sericea* | 108 | 91.4 | 35.9 |  | 122.7 |
| SRR27295752 | *Camarea axillaris* | 136 | 89.6 | 44.4 |  | 122.5 |
| SRR27295593 | *Galphimia gracilis* | 113 | 93.8 | 31.9 |  | 121.5 |
| SRR27295562 | *Galphimia glandulosa* | 97 | 95.7 | 33.3 |  | 121.1 |
| SRR27295535 | *Hiptage elliptica* | 113 | 94.7 | 39.1 |  | 120.7 |
| SRR27295665 | *Dicella macroptera* | 269 | 95.5 | 36.4 |  | 120.4 |
| SRR27295648 | *Bunchosia paraguariensis* | 199 | 96.1 | 34.9 |  | 120.4 |
| SRR27295763 | *Carolus chasei* | 180 | 91.4 | 38.1 |  | 117.3 |
| SRR27295591 | *Hiptage benghalensis* | 94 | 92.8 | 37.1 |  | 117.0 |
| SRR27295615 | *Stigmaphyllon bonariense* | 465 | 90.3 | 40.2 |  | 117.0 |
| SRR27295756 | *Blepharandra hypoleuca* | 160 | 81.6 | 38.0 |  | 116.1 |
| SRR27295645 | *Bronwenia ferruginea* | 115 | 95.7 | 37.3 |  | 115.9 |
| SRR27295604 | *Diplopterys heterostyla* | 131 | 93.9 | 36.6 |  | 114.7 |
| SRR27295554 | *Stigmaphyllon emarginatum* | 617 | 92.7 | 59.2 |  | 113.8 |
| SRR27295655 | *Galphimia australis* | 160 | 82.4 | 35.3 |  | 113.7 |
| SRR27295578 | *Aspidopterys cavaleriei* | 121 | 95.1 | 35.9 |  | 113.3 |
| SRR27295739 | *Stigmaphyllon puberum* | 621 | 91.0 | 39.8 |  | 109.8 |
| SRR27295647 | *Bronwenia acapulcensis* | 125 | 95.3 | 38.4 |  | 108.6 |
| SRR27295559 | *Stigmaphyllon paralias* | 606 | 91.5 | 41.7 |  | 108.2 |
| SRR27295551 | *Carolus renidens* | 109 | 93.1 | 41.0 |  | 107.5 |
| SRR27295736 | *Stigmaphyllon puberum* | 652 | 88.8 | 42.7 |  | 105.1 |
| SRR27295601 | *Mascagnia eggersiana* | 165 | 95.7 | 39.4 |  | 104.4 |
| SRR27295556 | *Stigmaphyllon emarginatum* | 845 | 90.1 | 41.8 |  | 104.3 |
| SRR27295543 | *Stigmaphyllon puberum* | 698 | 91.8 | 38.3 |  | 104.0 |
| SRR27295757 | *Cottsia californica* | 175 | 89.4 | 39.6 |  | 103.5 |
| SRR27295783 | *Christianella mesoamericana* | 183 | 94.7 | 37.7 |  | 102.3 |
| SRR27295678 | *Carolus chlorocarpus* | 26 | 89.6 | 37.7 |  | 101.8 |
| SRR27295811 | *Triaspis hypericoides* | 76 | 92.6 | 35.8 |  | 100.8 |
| SRR27295582 | *Blepharandra angustifolia* | 135 | 76.2 | 39.3 |  | 100.7 |
| SRR27295610 | *Stigmaphyllon bonariense* | 374 | 92.0 | 49.9 |  | 100.4 |
| SRR27295794 | *Thryallis laburnum* | 220 | 95.7 | 37.4 |  | 97.1 |
| SRR27295772 | *Byrsonima macrophylla* | 469 | 87.3 | 45.4 |  | 96.4 |
| SRR27295689 | *Carolus anderssonii* | 46 | 91.2 | 38.4 |  | 96.1 |
| SRR27295572 | *Banisteriopsis quadriglandula* | 133 | 94.2 | 35.2 |  | 94.6 |
| SRR27295731 | *Galphimia radialis* | 109 | 95.2 | 30.9 |  | 94.2 |
| SRR27295574 | *Banisteriopsis arborea* | 622 | 93.5 | 36.6 |  | 93.9 |
| SRR27295779 | *Malpighiodes leucanthele* | 135 | 93.9 | 38.0 |  | 93.8 |
| SRR27295753 | *Aspicarpa harleyi* | 128 | 92.3 | 37.8 |  | 90.9 |
| SRR27295770 | *Dicella nucifera* | 525 | 95.0 | 41.5 |  | 89.2 |
| SRR27295605 | *Mascagnia tenuifolia* | 144 | 95.2 | 37.7 |  | 88.8 |
| SRR27295713 | *Stigmaphyllon paralias* | 153 | 85.5 | 49.3 |  | 86.0 |
| SRR27295534 | *Heteropterys aenea* | 216 | 66.4 | 43.4 |  | 83.4 |
| SRR27295764 | *Aspicarpa pulchella* | 90 | 90.7 | 38.3 |  | 83.0 |
| SRR27295751 | *Hiptage detergens* | 85 | 88.8 | 37.1 |  | 82.6 |
| SRR27295632 | *Camarea affinis* | 118 | 93.3 | 37.9 |  | 82.4 |
| SRR27295603 | *Bronwenia cinerascens* | 85 | 94.5 | 36.6 |  | 82.3 |
| SRR27295747 | *Tetrapterys anomala* | 131 | 59.5 | 44.8 |  | 82.3 |
| SRR27295618 | *Stigmaphyllon jatrophifolium* | 892 | 91.1 | 43.2 |  | 81.0 |
| SRR27295780 | *Diplopterys populifolia* | 165 | 95.2 | 39.5 |  | 78.7 |
| SRR27295602 | *Banisteriopsis harleyi* | 59 | 87.4 | 38.0 |  | 76.9 |
| SRR27295573 | *Heteropterys hypericifolia* | 132 | 94.8 | 43.0 |  | 74.5 |
| SRR27295801 | *Hiptage myrtifolia* | 89 | 95.4 | 38.8 |  | 72.4 |
| SRR27295761 | *Burdachia sphaerocarpa* | 158 | 92.7 | 39.9 |  | 71.9 |
| SRR27295642 | *Banisteriopsis stellaris* | 112 | 92.3 | 39.8 |  | 71.9 |
| SRR27295785 | *Burdachia prismatocarpa* | 1005 | 95.4 | 42.6 |  | 70.5 |
| SRR27295728 | *Stigmaphyllon lindenianum* | 703 | 91.5 | 41.9 |  | 65.1 |
| SRR27295762 | *Acmanthera latifolia* | 174 | 93.5 | 38.0 |  | 64.6 |
| SRR27295767 | *Heteropterys molesta* | 315 | 95.5 | 36.9 |  | 64.2 |
| SRR27295735 | *Stigmaphyllon lindenianum* | 1,010 | 91.2 | 42.8 |  | 64.0 |
| SRR27295641 | *Bronwenia wurdackii* | 127 | 95.6 | 38.4 |  | 63.7 |
| SRR27295609 | *Malpighiodes guianensis* | 137 | 92.3 | 54.6 |  | 62.1 |
| SRR27295637 | *Stigmaphyllon ciliatum* | 265 | 87.6 | 45.4 |  | 59.5 |
| SRR27295640 | *Diplopterys pubipetala* | 137 | 95.1 | 37.9 |  | 53.9 |
| SRR27295748 | *Heteropterys pteropetala* | 331 | 95.5 | 52.8 |  | 52.7 |
| SRR27295537 | *Bunchosia pilocarpa* | 492 | 94.5 | 33.9 |  | 47.3 |
| SRR27295793 | *Acmanthera cowanii* | 194 | 72.8 | 44.4 |  | 45.1 |
| SRR27295789 | *Aspidopterys cordata* | 18 | 70.7 | 42.4 |  | 33.3 |
| SRR27295769 | *Heteropterys riparia* | 494 | 95.9 | 39.7 |  | 30.2 |
| SRR27295730 | *Stigmaphyllon lindenianum* | 443 | 90.1 | 45.8 |  | 29.3 |
| SRR27295581 | *Bunchosia postuma* | 142 | 94.7 | 33.1 |  | 29.0 |
| SRR27295727 | *Stigmaphyllon lindenianum* | 917 | 91.1 | 43.4 |  | 28.9 |
| SRR27295802 | *Diacidia aracaensis* | 34 | 81.1 | 40.0 |  | 27.6 |
| SRR27295617 | *Stigmaphyllon bonariense* | 627 | 91.3 | 41.0 |  | 26.9 |
| SRR27295627 | *Stigmaphyllon jatrophifolium* | 377 | 91.5 | 40.6 |  | 23.9 |
| SRR27295583 | *Byrsonima viminifolia* | 262 | 95.4 | 36.8 |  | 21.4 |
| SRR27295550 | *Stigmaphyllon emarginatum* | 318 | 88.6 | 46.2 |  | 12.2 |
| SRR27295552 | *Stigmaphyllon emarginatum* | 91 | 87.9 | 59.6 |  | 5.4 |
| SRR27295676 | *Stigmaphyllon ciliatum* | 1,446 | 89.6 | 46.0 |  | 3.0 |
| SRR27295635 | *Stigmaphyllon ciliatum* | 91 | 75.2 | 55.2 |  | 2.9 |
| SRR27295673 | *Stigmaphyllon bannisterioides* | 34 | 75.1 | 71.0 | failed | 0.0 |
